# Class-Hidden Client-Side Watermarking in Federated Learning

**DOI:** 10.3390/e27020134

**Published:** 2025-01-27

**Authors:** Weitong Chen, Chi Zhang, Wei Zhang, Jie Cai

**Affiliations:** 1 School of Information Engineering, Yangzhou University, Yangzhou 225009, China; wtchen@yzu.edu.cn (W.C.); mz120241049@stu.yzu.edu.cn (C.Z.); caijie@yzu.edu.cn (J.C.); 2 Jiangsu Province Engineering Research Center of Knowledge Management and Intelligent Service, Yangzhou 225127, China

**Keywords:** federated learning, intellectual property protection, model watermarking, watermark class

## Abstract

Federated learning consists of a central aggregator and multiple clients, forming a distributed structure that effectively protects data privacy. However, since all participants can access the global model, the risk of model leakage increases, especially when unreliable participants are involved. To safeguard model copyright while enhancing the robustness and secrecy of the watermark, this paper proposes a client-side watermarking scheme. Specifically, the proposed method introduces an additional watermark class, expanding the output layer of the client model into an N+1-class classifier. The client’s local model is then trained using both the watermark dataset and the local dataset. Notably, before uploading to the server, the parameters of the watermark class are removed from the output layer and stored locally. Additionally, the client uploads amplified parameters to address the potential weakening of the watermark during the aggregation. After aggregation, the global model is distributed to the clients for local training. Through multiple rounds of iteration, the saved watermark parameters are continuously updated until the global model converges. On the MNIST, CIFAR-100, and CIFAR-10 datasets, the watermark detection rates on VGG-16 and ResNet-18 reached 100%. Furthermore, extensive experiments demonstrate that this method has minimal impact on model performance and exhibits strong robustness against pruning and fine-tuning attacks.

## 1. Introduction

Federated learning [1,2] is a distributed machine learning method that effectively addresses data privacy concerns and alleviates problems caused by data siloing [3,4,5]. However, the performance of such models comes at the cost of an expensive training process, requiring substantial training data, specialized hardware, and infrastructure. Federated learning is commonly applied in sensitive domains, such as healthcare and finance, where the security of the model directly impacts individuals’ health and financial well-being. Nevertheless, allowing all clients access to the global model in federated learning increases the risk of model leakage [6,7,8]. Therefore, protecting the copyrights of federated learning models is of paramount importance; otherwise, it may lead to business malicious competition, knowledge theft, or other malicious purposes [9,10,11,12].

To protect the intellectual property of deep learning models [13,14], scholars have introduced the concept of model watermarking [15,16,17]. Model watermarking involves embedding unique identifiers or features into the parameters, structure, or outputs of the model to mark ownership information. If the model is illegally copied or stolen, the watermark can help identify the copyright. Additionally, watermarking techniques can be combined with other security measures such as model encryption, access control, etc., to further enhance the security and copyright protection of the model. Currently, watermarking techniques are divided into white-box watermarking [18,19,20] and black-box watermarking [21,22,23]. White-box watermarking involves accessing the model’s internal parameters and embedding the watermark by modifying parameters or altering the model structure. The verification process also requires access to internal information [24,25]. White-box watermarks typically offer higher robustness and security because the watermark information is directly embedded into the model’s internal structure. However, since access to the model’s internal information is required, white-box watermarking may introduce certain computational and storage overheads, and may potentially leak some sensitive information about the model. On the other hand, black-box watermarking does not require access to the model’s internal information during embedding and verification processes. It embeds watermark information into the model’s input and output behaviors, relying solely on specific model outputs for watermark validation. Therefore, black-box watermarking is generally more lightweight. However, black-box watermarks may be more vulnerable to attacks, as the watermark information relies solely on the model’s output behavior, which may be subject to external interference or attacks.

Although model watermarking techniques have been widely applied in various fields, their application in federated learning models still faces numerous challenges. Federated learning, as a distributed learning framework, involves participants training models locally, with the central server unable to directly access the data. This architecture introduces new issues, such as watermark attenuation after aggregation, aggregation failures due to model structure changes (structure-based white-box watermarking), and impacts on the accuracy of the original task (black-box watermarking). These challenges make watermarking techniques designed for centralized learning unsuitable for federated learning. To address these challenges, researchers have proposed watermarking algorithms specifically designed for federated learning models. These algorithms are generally classified into two types: server-side watermarking [26,27] and client-side watermarking [28,29]. Server-side watermarking is intended to protect the global model, which is collaboratively trained by participants and aggregated on the central server. It embeds specific markers or features to identify the ownership of the model, preventing unauthorized replication. Client-side watermarking is used for ownership verification or tracking, preventing unauthorized copying or theft of the model. This watermarking is embedded during the local training process on participants’ devices, with the server not needing to directly access the training data.

These model watermarking methods have been adjusted and optimized in both white-box and black-box watermarking techniques to adapt to federated learning scenarios. However, white-box federated model watermarking algorithms require modifying the model weights and training based on specific loss functions to embed watermark information. While this approach enhances the robustness of the watermark, it significantly increases the algorithm’s complexity and training overhead. In contrast, black-box federated model watermarking algorithms embed triggers into the original task data, allowing the model to learn the mapping between the triggers and specific classes in the original task. Due to the significant differences between the trigger features and the original task features, this inevitably impacts the accuracy of the original task, particularly negatively affecting the accuracy of the classes containing the watermark samples.

To address the aforementioned issues, we propose an innovative watermarking algorithm that ensures a high watermark verification success rate while minimizing the impact on the original task, making it almost negligible, and avoiding an increase in model complexity. This approach adds an additional “watermark” class to the training data instead of embedding a watermark behavior directly into a specific class of the original task, thereby preventing interference with the classification task. This method makes the watermark more covert and difficult to detect while not affecting the model’s original task. Specifically, we assume that the initiator’s client is trustworthy. First, we create a private watermark dataset and modify the model structure from an N-class classification model to an (N + 1)-class classification model. During the client’s training process, the watermark is embedded into the model. Before submitting the model for aggregation, the client restores the original model structure and retains the output layer parameters related to the watermark class, continuously updating these parameters. Meanwhile, untrustworthy clients focus solely on the original task. During verification, the stored watermark parameters are reintegrated into the model’s output layer to confirm the ownership of the federated learning model. Additionally, we introduce a scaling factor ξ to address issues of aggregation failure and watermark attenuation. The main contributions of our work include the following:

• This study proposes a watermarking method that ensures watermark labels are independent of the primary task labels, thereby avoiding interference between watermark information and task outputs. This independence ensures that watermark embedding does not affect the performance of the model’s primary task, guaranteeing high fidelity.
• By modifying the model’s structure, the watermark can be effectively embedded into different types of models without relying on any specific class, thereby enhancing the adaptability of the watermark.
• Various experiments demonstrate that the proposed method has strong robustness against pruning and fine-tuning attacks.

The remainder of this paper is structured as follows: Section 2 provides an overview of existing deep learning and federated learning watermarking technologies. Section 3 presents a general description of the threat model and the requirements for watermarks. The proposed method and algorithm are discussed in Section 4. Section 5 evaluates and analyzes the experimental results. Finally, Section 6 concludes this work.

## 2. Related Works

### 2.1. Centralized and Federated Learning Watermarking Schemes

White-box Watermarking Schemes: White-box watermarking refers to the method of directly embedding a watermark into a model by modifying its weights or structure. In centralized learning, white-box watermarking schemes are mainly categorized into weight-based [30,31,32,33] and structure-based watermarking [19,34,35]. In weight-based watermarking schemes, Uchida et al. [31] were the first to propose a method of embedding a watermark into a DNN model by imposing an additional regularization term on the weight parameters. Later, Liu et al. [32] introduced a method to enhance watermark robustness by selecting fewer but important parameters for embedding the watermark while introducing regularization terms. Additionally, Yan et al. [33] proposed a method of injecting virtual neurons into the model to resist neural structure obfuscation. In these weight-based white-box watermarking schemes, access to the internal information of the DNN is required during the validation phase to detect the watermark.

In structure-based watermarking schemes, methods such as in [19] use passports for deep neural network ownership verification, where using incorrect passports significantly degrades the performance of the original task. Zhang et al. and Fan et al. [34,35] improved this approach, enhancing the watermark’s robustness against deletion attacks and ambiguity. Although structural watermarking schemes have certain advantages, such as being independent of model parameters and offering enhanced resistance to deletion attacks, they also come with drawbacks and challenges that need to be carefully considered and balanced in practical applications.

Black-box Watermarking Schemes: Black-box watermarking typically uses specific trigger samples to embed unique watermark information to verify model ownership. Adi et al. [36] embedded a watermark into the model using a trigger set made up of predefined abstract samples known only to the model owner. Li et al. [37] generated trigger construction algorithms using frequency-domain image watermarking and verified ownership of remote suspicious models by issuing trigger queries to them. Similarly, Pyone et al. [18] employed learnable transformational images to embed watermark patterns into the model, enabling remote verification of model ownership. Since black-box watermarks generally rely only on the model’s output features or markers, attackers may confuse or destroy the watermark by forging similar features, thus complicating watermark verification.

### 2.2. Federated Learning Watermarking Schemes

Server-side Watermarking Schemes: In federated learning, server-side watermarking typically involves embedding the watermark into the global model after parameter aggregation, which is then distributed to the clients [26,27,38,39]. Merkle-Sign [40] uses Merkle trees [41] to verify ownership, suitable for collaborative client-server environments. The server embeds client and server keys in each round, uploads them to the Merkle tree, and eventually updates all client keys. FedTracker [42] employs a dual-layer protection scheme, including a global watermark mechanism and a local fingerprint mechanism, respectively providing ownership verification and traceability. Furthermore, Chen et al. [27] introduced a detector to capture the relationship between the critical sample feature distributions of the target model’s output and critical sample labels, effectively verifying ownership based on the detector’s accuracy.

Client-side Watermarking Schemes: Client-side watermarking in federated learning is a technique used to verify model ownership [28,29,39,43], involving embedding specific identifiers or information into the client’s model during training to ensure the model’s ownership. The main difference from server-side watermarking lies in their embedding locations. Liu et al. [43] predesigned a set of noise patterns as watermarks embedded into client models, influencing the aggregation process of the global model. FedIPR [28] is designed such that each client can embed its private watermark, based on private features and backdoors, to independently assert ownership and verify the watermark. Li et al. [28] allow each client to embed its private watermark, which can be either white-box or black-box, to independently assert ownership and detect free riders. Liang et al. [29] utilize replaceable client-side watermarking techniques to update the federated learning model every few rounds, enabling each model to use a unique watermark.

## 3. Threat Model and Watermark Requirements

### 3.1. Threat Model

Our current work faces two primary threat models. Firstly, it aims to prevent unauthorized replication and misuse of the model by other clients. In such scenarios, malicious clients may illicitly acquire well-trained models and employ them for commercial competition, privacy infringement, or other unethical purposes. This could lead to intellectual property violations, harming the interests of the model owner, and potentially compromising the reliability and security of the entire federated learning system. Secondly, there is a risk of privacy breaches due to dishonest servers. These servers may attempt to steal model parameters during the training process to gain unfair advantages, or inadequate security measures during transmission or storage may lead to data leaks.

### 3.2. Watermarking Scenario and Requirements

In this work, we consider a scenario where *n* clients collaborate with an owner (*O*) to jointly train a deep learning model *f* to address complex pattern recognition tasks. It is important to note that the owner (*O*) is not only a participant in the training process but also the initiator of the model. As described in Section 3.1, potential attackers (*A*) may exist either among the participating clients or as external malicious users. To effectively resist potential attacks, our algorithm needs to possess the following characteristics:

Fidelity: In the field of digital watermarking, fidelity refers to whether the quality, accuracy, and integrity of the original data are effectively maintained after embedding the watermark.

Detectability: Watermark detectability denotes the ability to extract and successfully identify watermark information from a model that has embedded the watermark. A watermark with high detectability can be accurately discovered and extracted during the verification phase.

Robustness: Robustness is the most important characteristic of watermarking algorithms, and the watermark embedded in the model should have strong resistance to various attacks while maintaining the performance of the model.

Secrecy: A good watermark should be imperceptible, meaning that users or consumers should not be able to detect the watermark’s presence when using the model for predictions.

False negative ratio: The false negative ratio indicates the probability that watermark samples are incorrectly classified as clean categories during watermark verification. A lower false negative ratio signifies higher accuracy in watermark detection.

## 4. Proposed Method

This section presents the watermark dataset generation, watermark embedding, and detection procedures of the proposed watermarking scheme. During the process of generating the watermark dataset, we create a user-specific watermark, which is used to construct a user-specific, private watermark dataset. During the watermark embedding phase, the model is trained using both the watermark dataset and the local original dataset. A new watermark class is introduced in the output layer for training, achieving watermark embedding. Subsequently, restore the model structure, and the parameters of the watermark class are saved to ensure their secrecy and invisibility, thus enabling copyright traceability and identification. In the watermark detection phase, an independent third-party certification authority is responsible for generating the watermark dataset and verifying the ownership of the suspicious model using the results obtained from the watermark dataset.

### 4.1. Watermark Dataset Generation

**Figure 1:**
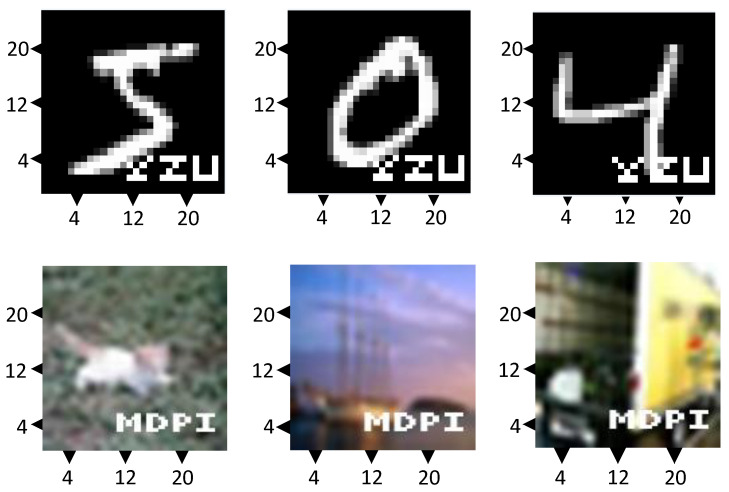
Samples in the watermark dataset.

The watermark dataset is constructed by randomly selecting a small number of samples from the training data. Our method involves randomly picking samples from each class. The creation of the watermark dataset includes selecting text or identifiers associated with identity information and embedding them into designated locations within the samples. In this work, the watermark is embedded in the lower right corner of the samples (Figure 1). The process can be mathematically expressed as follows:

(1) $$D_{w}\leftarrow \left\{W_{ij}=D_{ij}+P|0<i<C,j\in \left[0,Z\right]\right\}$$

Here, $D_{w}$ represents the watermark dataset, $W_{ij}$ refers to each sample in the watermark dataset, $D_{ij}$ denotes the original sample, and *P* signifies a particular pattern. In this equation, *i* is the class index, *j* indicates the sample index randomly selected from each class (with a selection of 5 samples), and *Z* denotes the maximum number of samples per class.

### 4.2. Watermark Embedding

The watermark dataset has its own labels, which are independent of the original dataset. Therefore, our expected goal during watermark embedding is that in the watermarked models, watermark samples are classified into the corresponding watermark classes, as described in Equation (2). In the clean models, since there are no classes corresponding to the watermark, watermark samples are classified based on content other than the watermark pattern, as described in Equation (3).

(2) $$\left\{\begin{array}{c} \tau_{c}=\delta_{w}\left(X_{c}\right)\\ \tau_{w}=\delta_{w}\left(X_{w}\right) \end{array}\right.$$

(3) $$\left\{\begin{array}{c} \tau_{c}=\delta_{c}\left(X_{c}\right)\\ \tau_{c}=\delta_{c}\left(X_{w}\right) \end{array}\right.$$

here, *X* and δ represent the input sample and the model, respectively. τ represents the predicted class of the sample by the model, with the subscripts *c* and *w* denoting clean (no watermark) and watermarked, respectively.

**Figure 2:**
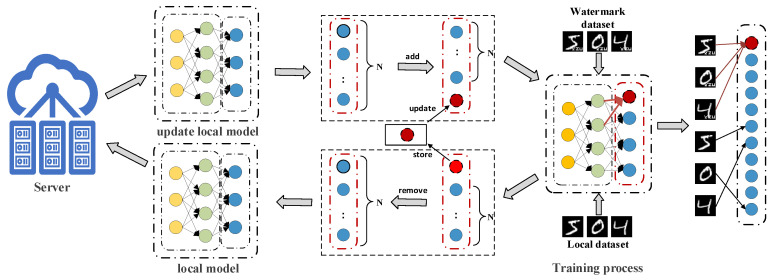
Watermark embedding process.

During the watermark embedding process, participants in the training are divided into two groups: the model initiator, referred to as client *O*, and the remaining clients (Figure 2). During the model training phase, the server and clients work with a shared model configured for *N* classifications. Under this framework, the other clients train solely on their local datasets without any additional operations. In contrast, client *O* trains on both the local dataset and the watermark dataset.

After training, the locally updated models from all clients are uploaded to the server for aggregation, and the aggregated model is distributed back to each client for the next training round. This process is detailed in Algorithm 1, which illustrates the steps of watermark embedding performed by the owner client and the subsequent server-side aggregation:

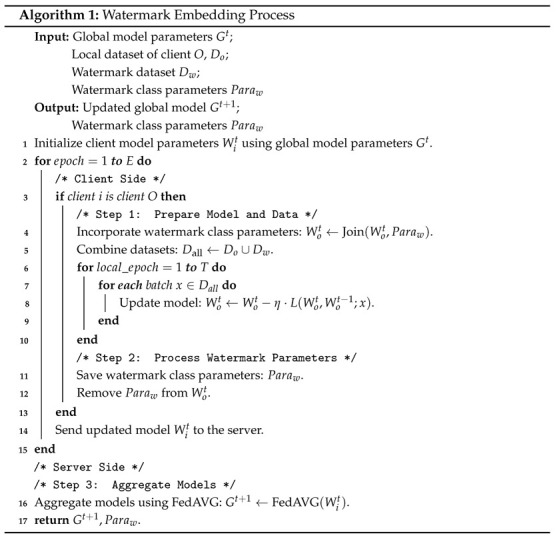


Step 1: As defined in Equation (4), during the local training rounds of client *O*, after receiving the model parameters $G_{i}$, client *O* modifies the model’s output layer from *N* classes to N+1 classes. Subsequently, the original task dataset is combined with the generated watermark dataset to form $D_{all}$.

(4) $$D_{all}=D_{o}+D_{w}$$

where $D_{o}$ represents the local dataset of client *O*, $D_{w}$ denotes the watermark dataset, and $D_{all}$ is the dataset composed of both. During the local training process, the selected loss function updates the parameters based on the dataset $D_{all}$ for local training of the model parameters. Equation (5) defines the loss function, which is consistent with the typical federated learning loss function.

(5) $$W_{o}^{\left(t+1\right)}=\arg\min_{W}\frac{1}{N_{o}}\sum_{\left(x_{oj},y_{oj}\right)\in D_{all}}L\left(W,x_{oj},y_{oj}\right)$$

where $W_{o}^{\left(t+1\right)}$ is the model parameters updated by participant *o* after *t* rounds of training. $N_{o}$ means the size of the local training dataset owned by participant *o*. *j* is the index of the dataset. $L(W,x_{oj},y_{oj})$ denotes the loss function used to quantify the discrepancy between the model’s output for the input sample $x_{oj}$ and its corresponding true label $y_{oj}$.

Step 2: After completing local training, the watermark parameters in the output layer are stored, and the model’s output is adjusted from N+1 classes back to *N* classes (as shown in Figure 2). This step aims to conceal the watermark class information and ensure that the local model of client *O* is consistent with the structure of the aggregated model. Since the model has no commercial value in the initial training stages, the watermark embedding begins when the model reaches commercial value (epoch = *e*) and continues until the end of training (epoch = *E*), as shown in Algorithm 1.

(6) $$\begin{array}{c} G^{\left(t+1\right)}=G^{t}+\frac{1}{m}\times \sum_{i=1}^{m}\left(W_{i}^{\left(t+1\right)}-G^{t}\right) \end{array}$$

(7) $$\begin{array}{c} G^{\left(t+1\right)}=G^{t}+\frac{\eta }{n}\times \sum_{i=1}^{m}\left(W_{i}^{\left(t+1\right)}-G^{t}\right) \end{array}$$

Additionally, during training, client *O* may not be selected for model aggregation in every iteration, meaning that the watermark information from its local training may not be sent to the server for aggregation every time. To address this issue, we introduce a scaling factor ξ [44] to amplify the client’s parameters. This is a widely used mechanism aimed at adjusting each client’s contribution to model updates, thereby balancing the differences in data volume and importance among clients.

(8) $$\begin{array}{c} \sum_{i=1}^{m-1}\left(W_{i}^{\left(t+1\right)}-G^{t}\right)\approx 0 \end{array}$$

(9) $$\begin{array}{c} W_{i}^{\left(t+1\right)}\approx \frac{n}{\eta }\times \left(G^{\left(t+1\right)}-G^{t}\right)+G^{t} \end{array}$$

Global model aggregation can be expressed by Equation (6). We modify it to Equation (7), where η represents the global learning rate. When $\eta =\frac{n}{m}$, these two equations are equivalent. Due to the nature of federated learning, the aggregation process gradually reduces the bias until balanced, as shown in Equation (8). Therefore, Equation (7) can be simplified to Equation (9), where $\xi =\frac{n}{\eta }$, indicating that the weight $W_{i}^{\left(t+1\right)}$ is amplified by a factor of ξ.

(10) $$\begin{array}{c} \xi =\frac{Client_{all}}{Client_{sect}} \end{array}$$

As noted in [44], when $\frac{n}{\eta }=\frac{Client_{all}}{Client_{set}}$, smaller weights can be well preserved. Thus, in our training, we use Equation (10) and adopt the method in Equation (11) to amplify the watermark-containing weights by a factor of ξ [44], addressing the issue of the watermark not being aggregated in every iteration.

(11) $$\begin{array}{c} W_{o}^{t}=\xi \times W_{o}^{t} \end{array}$$

Here, $Client_{all}$ is the total number of clients participating in the training, and $Client_{sect}$ is the number of clients selected for aggregation in a particular iteration. $W_{o}^{t}$ represents the local model parameters of client *O* uploaded to the server, which are amplified by ξ before uploading. Subsequently, client *O* uploads the model to the server for further model aggregation and updating.

Step 3: In federated learning, model updates are performed by the server through the averaging of the local model parameters received from the clients. This process involves combining the parameters from each client’s local model to form a global model, which reflects the collective contributions of all participants. The model aggregation procedure is as follows:

(12) $$G^{\left(t+1\right)}=\sum_{i=1}^{N}\frac{D_{i}}{D}W_{i}^{\left(t+1\right)}$$

Here, $G^{\left(t+1\right)}$ represents the parameters of the global model at the (t+1)-th round, while $W_{i}^{\left(t+1\right)}$ refers to the parameters of the local model of the *i*-th participant at the same round. *D* and $D_{i}$ represent the entire dataset and the dataset of the *i*-th client, respectively. The aggregation of local model parameters from all participants is represented by the summation symbol ∑ in the formula.

By iterating through steps 1 to 3, the model undergoes iterative training and watermark embedding until it is aggregated and the embedded watermark can be reliably extracted, obtaining the final watermarked model. This process not only ensures the effective preservation and utilization of watermark information but also optimizes the N+1 classification model. During local training, we train watermark datasets so that the model performs watermark embedding while maintaining the performance of the original task.

### 4.3. Watermark Verification

**Figure 3:**
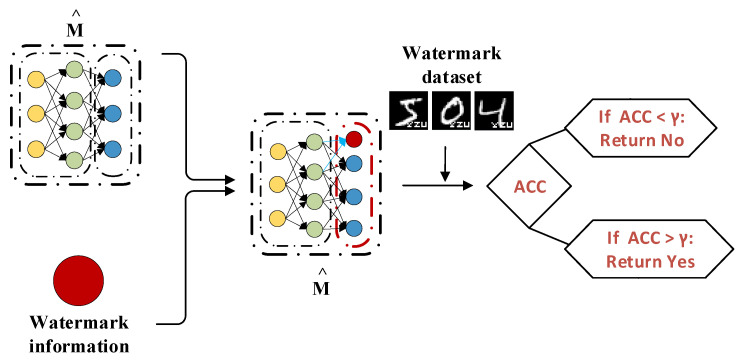
Watermark verification process.

If a suspected organization uses the model $\hat{M}$ without authorization from the model owner *O*, the owner has the right to involve an independent third-party certification agency to confirm the model’s copyright and produce relevant documentation. The watermark verification procedure is illustrated in Figure 3 and detailed in Algorithm 2. The third-party agency can carry out the watermark verification by following these steps:

Step 1: The model owner provides parameters $Para_{W}$ and watermark patterns, and third-party organizations can randomly select samples to generate watermark verification sets.

Step 2: The trained watermark class parameters $Para_{W}$ provided by the model owner are added into the output layer of the target model $\hat{M}$.

Step 3: Use the suspected model $\hat{M}$ to test the watermark dataset generated in Step 1. First, input the watermark samples into the target model $\hat{M}$ and record the model’s predictions for these samples. Based on the predicted results and the true labels of the watermark samples, calculate the prediction accuracy of the target model on the watermark samples. Specifically, the watermark class is considered private information of the model owner. Consistent watermark patterns are used throughout the training, testing, and validation processes. By determining whether the accuracy of watermark verification exceeds the predefined threshold γ, if the threshold is surpassed, it can be concluded that the target model $\hat{M}$ has been illegally stolen. The value of γ is primarily adjusted based on the size of the watermark set and the categories of the samples [20,36], and since the watermark set dynamically changes according to the size of the dataset and sample categories, γ is adjusted dynamically.

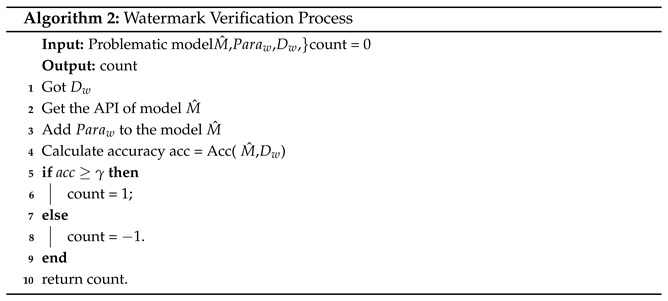


## 5. Experiments and Analysis

### 5.1. Experiment Settings

This study utilized the PyTorch 1.13.1 framework [45]. The hardware configuration included an NVIDIA GeForce RTX 4060 Ti GPU with 8 GB of memory, an Intel Core i5-13600KF CPU with 14 physical cores and 20 logical cores, and 31.82 GB of memory. The experiment involved 100 participants in a federated learning setup. The dataset was partitioned into 100 simulated client datasets based on independent and identically distributed (IID) samples, while under the non-independent and identically distributed (Non-IID) setting, the dataset was generated according to a Dirichlet distribution. The training process consisted of 250 communication rounds. In each round, the learning rate was set to 0.01, the momentum to 0.5, and the minibatch size to 32, with selected clients training locally for 10 epochs. In the comparative experiments, to ensure fairness, we applied the same parameter settings across all setups, and all experimental data were validated using the test set.

#### 5.1.1. Dataset and Model Settings

In this study, we used the MNIST, CIFAR-10, and CIFAR-100 datasets for experiments.

MNIST [46]: This dataset contains 60,000 28 × 28 grayscale images of handwritten digits and 10,000 test images, with about 6000 training images and 1000 test images per digit. Due to its simplicity, MNIST is commonly used for quick testing and comparison of image classification models.

CIFAR-10 [47]: CIFAR-10 comprises 60,000 color images across 10 categories, with 6000 images per category, each sized 32 × 32 pixels. The dataset includes diverse samples with varying angles, sizes, lighting conditions, and backgrounds, making it a standard benchmark for image classification tasks.

CIFAR-100 [48]: CIFAR-100 contains 60,000 images divided into 100 categories, with 600 images per category. Each image is 32 × 32 pixels, covering a broader range of objects and more complex content than CIFAR-10, suitable for testing deep learning models on more challenging tasks.

To evaluate our method, we selected two well-known network architectures: VGG-16 [49] and ResNet-18 [50]. ResNet-18 consists of an initial convolutional layer, four ResBlocks, and a fully connected layer, while VGG-16 includes 13 convolutional layers and 3 fully connected layers. Our method, applied to the fully connected layer, is compatible with most models.

#### 5.1.2. Watermark Dataset Setting

**Figure 4:**
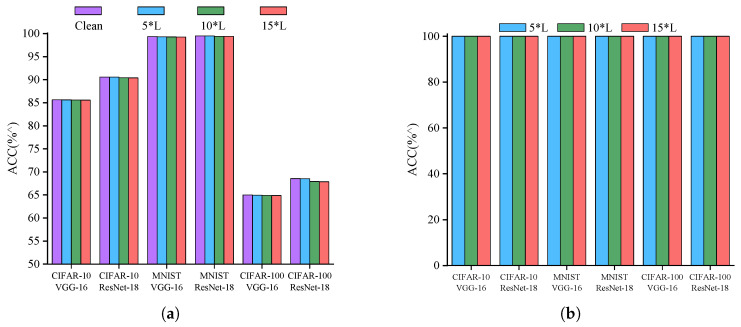
The impact of watermark dataset size: (**a**) The impact on the accuracy of the original task. (**b**) The impact on watermark detection rate.

To embed the watermark successfully without wasting computational resources, we conducted a series of experiments using 5, 10, and 15 samples per class to create the watermark dataset. We examined how the size of the watermark dataset affects the primary task and the accuracy of watermark detection. Assuming the original dataset contains L classes, the sizes of the watermark dataset are 5 L, 10 L, and 15 L. According to our experimental results, it can be known that the decline in the original task accuracy caused by the watermark dataset size varying from 5 L to 15 L is insignificant, with a maximum decrease of only 0.06% (Figure 4a). This indicates that the impact of the watermark dataset size on the main task can be considered insignificant. Additionally, the figures demonstrate that a watermark dataset of 5 L samples already achieves a 100% success rate in watermark embedding (Figure 4b). In summary, choosing a dataset of 5 L samples for watermarking can efficiently embed the watermark with minimal interference in the original task. Considering the idea of minimizing computational resources, we randomly select 5 samples from each class for subsequent experiments.

### 5.2. Performance Analysis Under IID

#### 5.2.1. Fidelity

A comparative analysis between the accuracy of the pristine model and the watermarked model was conducted on the test set. In the experiment, a total of 250 iterations were completed throughout the training phase. Given the absence of commercial significance in the initial stages of training, watermark embedding was deemed unnecessary. Consequently, in our experiments, watermark embedding commenced from the 100th iteration onwards.

**Figure 5:**
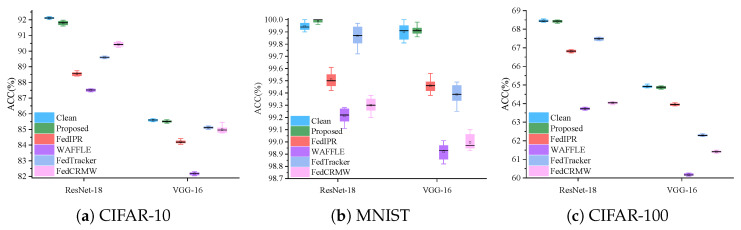
Comparison of original task accuracy.

We compared the proposed class-hidden scheme with the FedIPR [28], WAFFLE [38], FedTracker [42], and FedCRMW [51] schemes while evaluating the impact of watermark embedding on the original task accuracy (see Figure 5). To ensure that the experimental results are statistically significant and not due to random fluctuations, we conducted 100 rounds of experiments with the same parameters for each group. A comparison of accuracy before and after watermark embedding showed that the proposed scheme resulted in accuracy reductions of 0.15%, 0.13%, 0.09%, 0.07%, 0.12%, and 0.29%. In comparison, the FedIPR [28] scheme led to accuracy drops of 1.48%, 1.6%, 1.79%, 1.18%, 1.51%, and 1.43%; the WAFFLE [38] scheme showed accuracy decreases of 1.35%, 1.68%, 1.72%, 1.25%, 1.47%, and 1.55%; the FedTracker [42] scheme caused accuracy decreases of 1.93%, 2.91%, 1.8%, 1.73%, 1.28%, and 1.25%; and finally, the FedCRMW [51] scheme exhibited accuracy reductions of 1.31%, 1.62%, 1.65%, 1.22%, 1.44%, and 1.63%. Based on the results above, we can confirm that the experimental findings are statistically significant and not due to random variations and that the proposed method has the least impact on the original task. Therefore, we conclude that the proposed class-hidden embedding scheme successfully embeds watermark information while maintaining high accuracy on the original task.

#### 5.2.2. Detectability

A watermark dataset is first constructed to verify the detectability of the model watermark. Subsequently, during the training of the local model on client *O*, the parameters associated with watermark information in the output layer were continuously updated and saved. Finally, the detectability of the model is evaluated by calculating the watermark detection rate of the global model.

**Figure 6:**
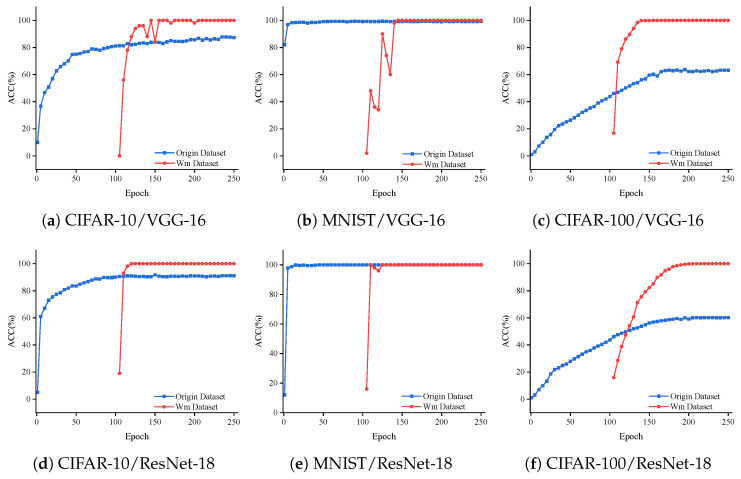
Accuracy of original task and watermark across different training epochs.

As shown in Figure 6, it demonstrates the accuracy of the original tasks and watermark detection when embedding the watermark for the ResNet-18 and VGG-16 models on the MNIST, CIFAR-10, and CIFAR-100 datasets. For the VGG-16 model, the watermark detection accuracy is above 80% after 40 iterations and remains at 100% after 60 iterations (Figure 6a,b). We observe that the watermark detection accuracy on the ResNet-18 model surpasses 95% for the MNIST, CIFAR-10, and CIFAR-100 datasets after 10 iterations and remains at 100% after 25 iterations (Figure 6d,e). Our proposed method demonstrates that after embedding the watermark, the accuracy of the primary task remains unaffected, indicating that our approach maintains a high watermark detection capability.

#### 5.2.3. Robustness

Watermark robustness refers to the capacity of the watermark to stay detectable and recognizable under various attacks and processing conditions. In this paper, we evaluated the robustness of the watermark by testing its resilience against fine-tuning and pruning attacks.

**Figure 7:**
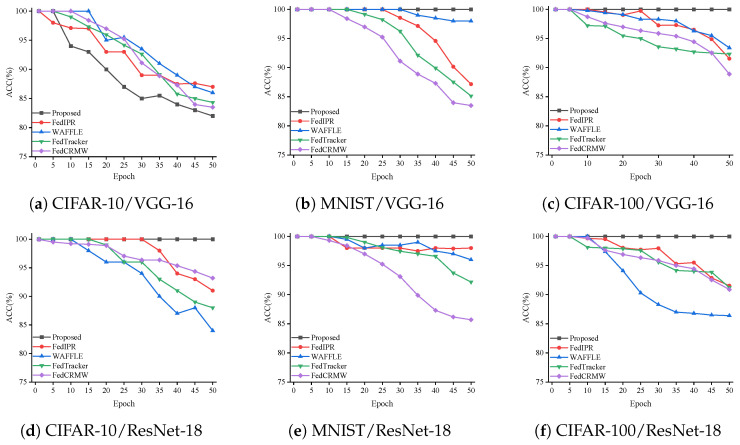
Comparison of watermark accuracy across fine-tuning epochs.

**Fine-tuning Attack**: Fine-tuning refers to the additional training of a pretrained model using an additional dataset to adapt it to a new task. This process is achieved by making small adjustments and optimizing the parameters of the existing model. In the experiment, we performed fine-tuning with a dataset that excluded watermark samples, and no parameters of any layer were frozen. Figure 7 shows the changes in the watermark detection rates of our proposed method compared with those of the other three methods after 50 fine-tuning iterations. In the CIFAR-10/VGG-16 experiment, we set the scaling factor ξ to the predefined value of 50%. As can be clearly seen from Figure 7, with this setting, watermark information is easily lost during the fine-tuning process, resulting in our method having a slightly lower watermark detection rate compared with FedIPR [28], WAFFLE [38], and FedCRMW [51]. However, in the other five experiments, we used the predefined ξ, further validating the conclusions in [44]. The final results show that our method consistently outperforms FedIPR [28], WAFFLE [38], and FedCRMW [51], maintaining a 100% detection rate. Overall, our method demonstrates good performance during the fine-tuning process.

**Pruning Attack**: Pruning attacks involve intentionally removing or disrupting embedded watermark information in a model by applying pruning operations. In this study, we utilized a weight-based pruning method to modify the model. Table 1 presents a comparison of the robustness of the VGG-16 model under pruning attacks. It can be observed that as the pruning ratio increases, the accuracy of all watermark schemes on the original task decreases. However, compared with the methods of FedIPR [28], WAFFLE [38], FedCRMW [51], and FedTracker [42], our proposed watermarking scheme demonstrates a smaller reduction in accuracy on the original task. Moreover, even with a pruning ratio as high as 90%, our method maintains a watermark detection rate of 100%. Overall, under the same pruning ratio, our scheme exhibits stronger resilience to pruning attacks.

**Figure 8:**
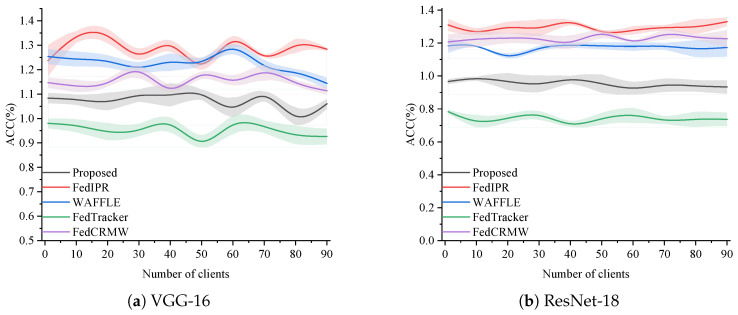
Comparison of original task accuracy against forging attack.

**Forging Attack**: A forging attack is a security threat where attackers fabricate data or images to bypass security defenses. In this context, attackers may have pinpointed the location where the watermark is embedded in our model. Consequently, they could attempt to forge additional parameter weights to replace the original ones, aiming to bypass the watermark and illicitly use the model. In our experiment, we tested two models on CIFAR-10, using 90 clients for each model, with the number of clients gradually increasing. The final results, shown in Figure 8, reveal that after the weights were replaced, the model’s accuracy plummeted to about 1%. The accuracy of FedIPR [28], WAFFLE [38], FedTracker [42], and FedCRMW [51] also dropped to 1%. This accuracy indicates that the model becomes non-functional after the weight replacement, making it commercially useless, and further demonstrates the robustness of our method in resisting forging attacks.

**Overwriting Attack**: The overwriting attack involves feeding the model a large amount of forged data, aiming to overwrite or replace the originally embedded watermark or identifying features, thereby preventing the model from correctly recognizing or verifying the original watermark. For the method proposed in this paper, malicious clients may input numerous other watermark features in an attempt to overwrite the model’s features, leading to a decrease in the model’s watermark accuracy. Table 2 presents the accuracy of our model’s private watermark after being overwritten by 1 to 10 ambiguous watermarks. The results show that although the model’s watermark accuracy decreases after overwriting, the impact is almost negligible, with the accuracy remaining above 99%. However, the proposed method also has certain limitations, notably the inability to identify the uniqueness of watermarks. While the accuracy of the private watermark does not significantly decline, the model is also capable of verifying the ambiguous watermarks used for overwriting.

#### 5.2.4. Secrecy

The proposed scheme combines the watermark dataset with client *O*’s local dataset for training. Before aggregation, the watermark class parameters are removed from the model’s output layer and stored locally, ensuring that the watermark dataset associated with the watermark class remains on the client side and is not shared with other participants. Additionally, the labels of the watermark dataset belong to independent categories outside the predefined labels. If client *O* does not provide the watermark parameters in the output layer, predictions on the watermark dataset will use its original semantic information labels. Only when the watermark dataset and the trained output layer watermark parameters are correctly provided, can the model output the correct labels. For other clients or observers who do not have the watermark class parameters, the model’s predicted labels for the watermark dataset will correspond to its original semantic information, making it impossible to infer the existence of the watermark.

**Figure 9:**
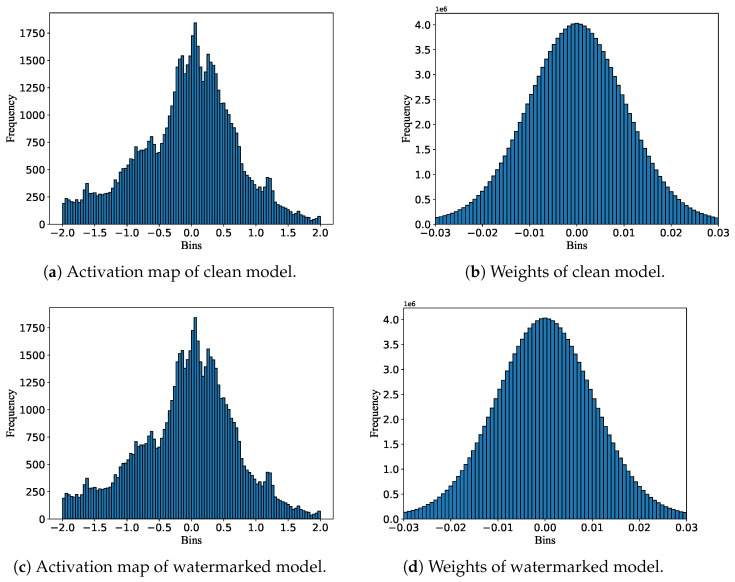
The activation maps and weight distributions of the watermarked model and the clean model on VGG-16.

As shown in Figure 9, the activation maps and weights of the watermarked model are identical to those of the clean model, demonstrating that the proposed watermarking scheme exhibits strong secrecy, making it difficult for attackers to perceive the presence of a watermark in the model.

#### 5.2.5. False Negative Ratio

**Figure 10:**
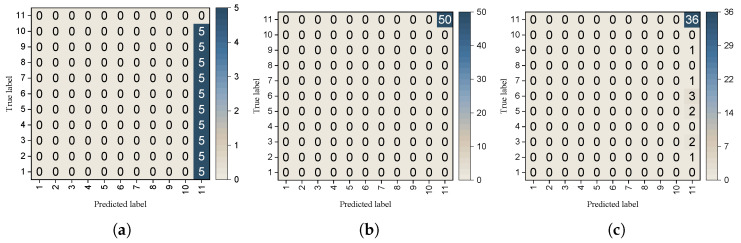
Distribution of prediction results for watermark datasets on clean and watermarked models: (**a**) Clean model. (**b**) Unmodified watermarked model. (**c**) Modified watermarked model.

The false negative rate refers to the probability that watermark samples are predicted as non-watermark categories during watermark verification. To evaluate the false negative rate, we separately assessed watermark verification on the CIFAR-10 dataset using both a clean ResNet-18 model and a ResNet-18 model embedded with watermarks. The results show that the clean model accurately classified watermark samples into their original semantic categories (Figure 10a). For models containing watermarks, the false negative rate can be categorized into two scenarios. The first scenario involves unaltered models with embedded watermarks, where the watermark dataset detection rate is 100% (Figure 10b), and the false negative rate is 0. The second scenario involves models with watermarks that have undergone some pruning or fine-tuning; while the detection accuracy of watermark samples may decrease, the watermark can still be detected (Figure 10c). In summary, whether in a clean model, a model embedded with watermarks, or a model subjected to attacks after watermark embedding, the false negative rate of the watermark proposed in this paper is very low, and its accuracy is sufficient to support copyright verification.

Additionally, we conducted watermark verification experiments on different types of samples, including original watermark samples, clean samples, and ambiguous watermark samples, using VGG-16 and ResNet-18 models. To enhance the credibility of the experimental results, we conducted 90 rounds of experiments, averaging every 10 rounds. As shown in Table 3, the verification success rate for original watermark samples reached 100%, the success rate for clean samples was 0%, and the success rate for ambiguous watermark samples was around 65%. Based on these results, it can be effectively demonstrated that the proposed method has a high watermark verification success rate.

#### 5.2.6. Efficiency Evaluation

We compared the efficiency of our method with recent white-box watermarking algorithms on the VGG-16 model. As shown in Table 4, the global model’s training time increased by only 0.13 s, 0.07 s, and 0.08 s, indicating that our watermarking approach added virtually no extra training burden during a single global training round.

In contrast, white-box methods such as FedTracker [42], FedIPR [28], and FedCRMW [51] employed more complex watermark embedding strategies, which resulted in additional time for embedding and training watermarks during the global model training process. Specifically, FedTracker [42] increased training time by 57.31 s, 56.71 s, and 37.07 s across the three datasets, FedIPR [28] added 104.04 s, 115.8 s, and 108.85 s, and FedCRMW [51] increased it by 67.74 s, 60.12 s, and 52.70 s, respectively.

In terms of verification efficiency, our method is comparable to other algorithms, requiring only 0.064 milliseconds per verification, while FedIPR [28] requires approximately 0.09 milliseconds, FedCRMW [51] 0.077 milliseconds, and FedTracker [42] 0.081 milliseconds.

The experimental results clearly demonstrate that our method achieves higher verification efficiency with minimal training overhead, highlighting its notable advantages.

### 5.3. Performance Analysis Under Non-IID

In federated learning, the non-independent and identically distributed (Non-IID) nature of client data is a common issue. Specifically, the datasets held by each client exhibit significant heterogeneity, with notable differences in labels and sample sizes within their training sets. Non-IID data negatively impact the effectiveness of federated learning. Given that Non-IID scenarios are more prevalent in real-world applications, experiments under such conditions are more meaningful.

**Figure 11:**
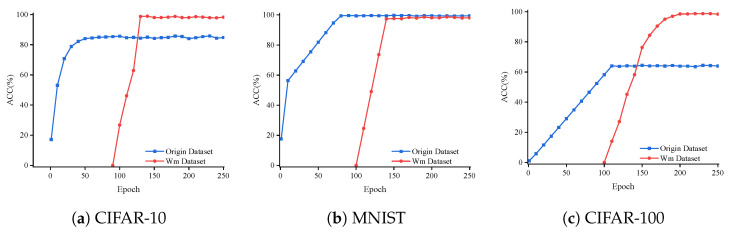
Accuracy of original task and watermark under Non-IID conditions.

We conducted experiments under Non-IID conditions using CIFAR-10, MNIST, and CIFAR-100 datasets, with VGG-16 as the chosen model. As shown in Figure 11, our method not only maintained the original task accuracy but also slightly improved it, with the watermark accuracy remaining around 98%. This demonstrates that our method remains effective even under Non-IID conditions.

#### 5.3.1. Robustness Against Fine-Tuning Attack

**Figure 12:**
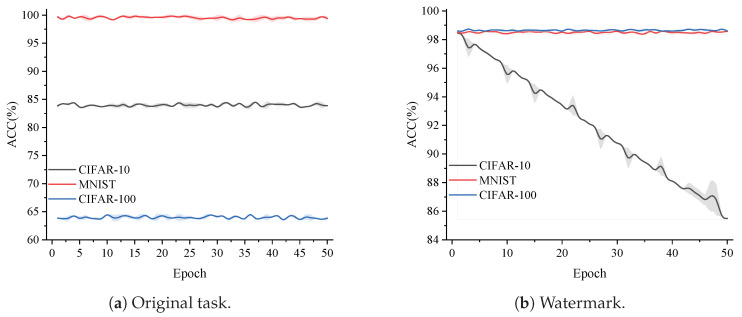
Accuracy of original task and watermark against fine-tuning under Non-IID conditions.

Similar to fine-tuning in the IID setting, we fine-tuned the client’s model using the client’s own dataset. The key difference is that, in the Non-IID setting, the data from different categories in the client’s dataset are imbalanced. The experimental results are shown in Figure 12.

As shown in Figure 12a, after 50 rounds of fine-tuning, the accuracy of the original task did not change significantly, with an overall variation of about 0.3%. From Figure 12b, it can be observed that, except for the CIFAR-10 dataset, where the watermark accuracy dropped to 85.4%, likely due to severe class imbalance in the client’s training data causing the model to fall into a local optimum, the watermark accuracy for the other two datasets did not show any noticeable change and remained above 98%. This conclusion is consistent with the results in the IID setting, indicating that after fine-tuning, our model is still able to perform well enough to support the verification task.

#### 5.3.2. Robustness Against Pruning Attack

**Figure 13:**
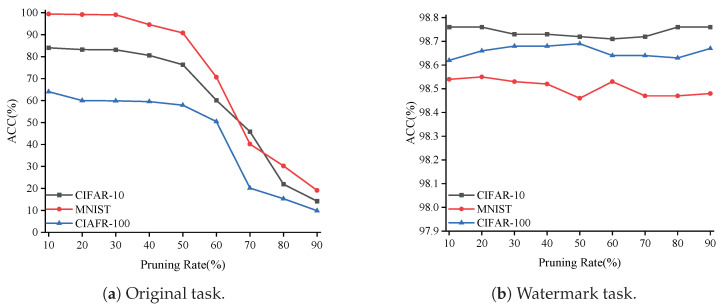
Accuracy of original task and watermark against pruning under Non-IID conditions.

We also conducted pruning experiments on the model under the Non-IID setting, with the pruning rate gradually increasing from 10% to 90%. As shown in Figure 13a, the accuracy of the original task does not significantly decrease until the pruning rate exceeds 60%. Figure 13b clearly shows that even when the pruning rate reaches 90%, our watermark accuracy remains above 98%. This strongly demonstrates that, even under Non-IID conditions, our model retains high robustness against pruning attacks.

#### 5.3.3. Robustness Against Overwriting Attack

**Figure 14:**
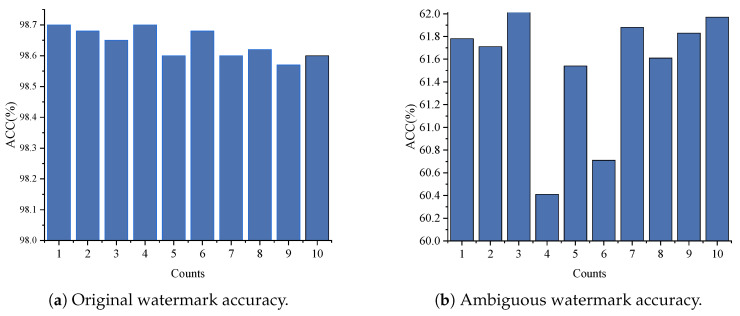
Watermark accuracy against overwriting attack under Non-IID conditions.

To evaluate whether the vulnerability of the watermark increases under the Non-IID condition, we conducted overwriting attack experiments on the model using the same attack setup as in the IID setting. The results are shown in Figure 14a. Whether applying one or ten ambiguous watermarks, our model still maintained an accuracy of over 98%. Although the model performs excellently in terms of watermark accuracy, unfortunately, it cannot achieve uniqueness. Additionally, we conducted an ambiguous watermark detection test, and as shown in the right-side of Figure 14b, the accuracy of each ambiguous watermark reached up to 60%, highlighting a limitation of our approach.

## 6. Conclusions

This paper introduces a class-hidden watermarking scheme to mitigate the impact of black-box watermarking on the accuracy of the original task in federated learning. The method modifies the local model structure during client training by adding a watermark class to embed the watermark. To maintain stealth, the parameters of the watermark class are hidden when uploading the model parameters. Experimental results show that, whether in IID or Non-IID scenarios, after embedding the watermark, the accuracy of the original classification task remains high, with the watermark’s impact on the original classification task being less than 0.3%, which is almost negligible. This scheme not only demonstrates good robustness, effectively resisting fine-tuning, pruning, forging, and overwriting attacks but also preserves the secrecy of the watermark. Therefore, in the context of federated learning, this scheme shows significant potential for practical applications, providing security guarantees for model safety in federated learning environments. Future work could focus on identifying malicious users, which would not only help with copyright verification but also enable the accurate tracking of leakage sources, identifying the specific client responsible for the leak. This may involve exploring user identification techniques and developing robust mechanisms to trace data leakage back to its origin, thus enhancing security measures in federated learning environments.

## Figures and Tables

**Table 1:** Accuracy of original task and watermark under different pruning rates on VGG-16.

Datasets	Evaluation Metrics	Methods	Pruning Rate								
			10%	20%	30%	40%	50%	60%	70%	80%	90%
cifar-10	Original Task Acc (%)	FedIPR	84.15	84.08	83.82	83.77	82.11	67.18	46.75	21.77	13.24
		WAFFLE	80.33	79.65	79.78	76.61	71.35	30.13	25.87	15.98	9.74
		FedCRMW	85.32	85.29	85.25	85.17	84.35	15.02	10.05	10.05	10.00
		FedTracker	85.12	84.97	84.88	84.86	76.33	25.05	15.81	11.83	6.17
		Proposed	84.95	84.90	84.82	84.80	84.64	84.59	64.55	29.19	15.91
	Watermark Acc (%)	FedIPR	100	100	100	100	100	54.12	32.15	15.42	10.37
		WAFFLE	100	100	98.55	98.55	80.31	80.33	76.15	66.41	32.98
		FedCRMW	98.41	98.41	98.41	98.41	98.41	15.87	6.35	6.35	6.35
		FedTracker	100	100	99.11	90.16	82.13	10.52	17.73	10.92	7.28
		Proposed	100	100	100	100	100	100	100	100	100
mnist	Original Task Acc (%)	FedIPR	99.56	99.46	99.44	99.31	99.12	98.92	65.72	32.13	15.17
		WAFFLE	99.15	99.12	99.04	98.99	97.21	76.52	32.13	15,91	10.11
		FedCRMW	98.81	98.81	98.8	98.35	91.48	22.15	11.35	11.35	11.35
		FedTracker	99.19	99.11	98.15	95.38	90.71	62.14	46.17	28.93	13.84
		Proposed	99.31	99.32	99.34	99.34	99.35	87.32	69.27	50.05	29.94
	Watermark Acc (%)	FedIPR	100	100	100	98.61	98.23	88.90	65.31	46.21	34.18
		WAFFLE	100	100	100	100	91.34	80.23	50.42	36.11	4.87
		FedCRMW	100	100	100	88.89	49.21	23.81	7.94	7.94	7.94
		FedTracker	100	100	100	95.17	51.84	35.96	21.75	17.96	6.92
		Proposed	100	100	100	100	100	100	100	100	100
cifar-100	Original Task Acc (%)	FedIPR	64.21	64.17	64.01	45.18	45.75	32.12	20.21	15.14	7.19
		WAFFLE	60.19	60.06	59.88	59.66	31.21	16.90	8.11	4.18	2.32
		FedCRMW	60.26	60.11	60.06	41.29	41.11	20.95	18.33	7.31	4.92
		FedTracker	64.41	64.19	63.16	51.83	43.73	26.18	13.84	9.17	5.73
		Proposed	64.55	64.55	63.17	63.07	40.15	20,84	16.31	14.29	8.59
	Watermark Acc (%)	FedIPR	100	100	100	96.19	96.14	60.42	54.86	32.18	15.81
		WAFFLE	100	100	100	100	49.16	31.58	19.28	4.13	0.00
		FedCRMW	100	100	100	79.15	48.92	37.95	20.73	15.75	6.95
		FedTracker	100	100	100	87.92	60.17	51.85	39.77	27.15	10.83
		Proposed	100	100	100	100	100	100	100	100	100

**Table 2:** Watermark accuracy under different numbers of ambiguous watermarks.

Methods	Models	Datasets	Number of Ambiguous Watermarks									
			1	2	3	4	5	6	7	8	9	10
Proposed	VGG-16	CIFAR-10	99.34	99.24	99.25	99.31	99.31	99.25	99.34	99.3	99.34	99.35
		MNIST	99.49	99.44	99.46	99.45	99.55	99.54	99.56	99.55	99.47	99.48
		CIFAR-100	99.08	99.06	99.14	99.04	99.16	99.13	99.11	99.05	99.10	99.05
	ResNet-18	CIFAR-10	99.53	99.44	99.46	99.51	99.54	99.56	99.45	99.5	99.5	99.48
		MNIST	99.63	99.63	99.6	99.57	99.55	99.62	99.59	99.65	99.59	99.64
		CIFAR-100	99.28	99.28	99.27	99.29	99.3	99.3	99.27	99.24	99.29	99.34
WAFFLE	VGG-16	CIFAR-10	65.11	65.12	65.09	65.08	65.13	65.14	65.1	65.14	65.14	65.11
		MNIST	70.08	70.08	70.13	70.15	70.08	70.1	70.13	70.09	70.16	70.06
		CIFAR-100	59.29	59.25	59.26	59.24	59.26	59.3	59.28	59.25	59.25	59.32
	ResNet-18	CIFAR-10	69.34	69.28	69.27	69.35	69.32	69.35	69.33	69.28	69.35	69.36
		MNIST	73.78	73.79	73.81	73.75	73.78	73.79	73.79	73.81	73.83	73.85
		CIFAR-100	62.78	62.78	62.77	62.81	62.77	62.81	62.83	62.83	62.85	62.82
FedIPR	VGG-16	CIFAR-10	87.65	87.66	87.65	87.71	87.66	87.71	87.74	87.7	87.67	87.73
		MNIST	89.75	89.75	89.75	89.75	89.66	89.65	89.74	89.67	89.69	89.71
		CIFAR-100	75.72	75.64	75.65	75.71	75.74	75.64	75.72	75.75	75.71	75.71
	ResNet-18	CIFAR-10	89.94	89.94	89.84	89.94	89.92	89.86	89.92	89.86	89.84	89.93
		MNIST	91.95	91.87	91.96	91.86	91.85	91.9	91.85	91.84	91.91	91.96
		CIFAR-100	80.95	80.91	80.85	80.89	80.9	80.92	80.88	80.89	80.86	80.95
FedCRMW	VGG-16	CIFAR-10	75.35	75.43	75.37	75.38	75.36	75.37	75.45	75.42	75.34	75.42
		MNIST	79.56	79.57	79.65	79.56	79.59	79.58	79.61	79.61	79.62	79.61
		CIFAR-100	71.61	71.64	71.62	71.59	71.65	71.62	71.63	71.63	71.57	71.58
	ResNet-18	CIFAR-10	87.56	87.65	87.65	87.63	87.54	87.58	87.56	87.65	87.63	87.6
		MNIST	85.16	85.05	85.1	85.1	85.13	85.12	85.11	85.14	85.1	85.12
		CIFAR-100	81.16	81.05	81.14	81.12	81.07	81.06	81.07	81.08	81.05	81.14
FedTracker	VGG-16	CIFAR-10	91.46	91.46	91.55	91.55	91.49	91.46	91.56	91.5	91.51	91.53
		MNIST	90.94	90.92	90.91	90.91	90.91	90.92	90.93	90.86	90.87	90.87
		CIFAR-100	93.85	93.84	93.91	93.85	93.86	93.87	93.9	93.87	93.89	93.92
	ResNet-18	CIFAR-10	96.12	96.06	96.08	96.15	96.07	96.06	96.16	96.08	96.08	96.15
		MNIST	95.68	95.76	95.67	95.71	95.67	95.69	95.76	95.68	95.65	95.76
		CIFAR-100	94.36	94.34	94.33	94.3	94.34	94.26	94.27	94.36	94.36	94.29

**Table 3:** Comparison of watermark accuracy with different types of samples.

Model	Acc(%)	Datasets	Number of Experiments								
			10	20	30	40	50	60	70	80	90
VGG-16	Watermark	CIFAR-10	100	100	100	100	100	100	100	100	100
		MNIST	100	100	100	100	100	100	100	100	100
		CIFAR-100	100	100	100	100	100	100	100	100	100
	Clean	CIFAR-10	0.00	0.00	0.00	0.00	0.00	0.00	0.00	0.00	0.00
		MNIST	0.00	0.00	0.00	0.00	0.00	0.00	0.00	0.00	0.00
		CIFAR-100	0.00	0.00	0.00	0.00	0.00	0.00	0.00	0.00	0.00
	Ambiguous	CIFAR-10	63.92	64.61	60.51	63.77	61.26	62.82	60.97	60.73	63.99
		MNIST	61.21	63.48	58.82	58.16	62.22	59.02	62.64	59.99	59.08
		CIFAR-100	66.09	63.84	65.23	64.24	63.43	66.81	65.01	65.04	65.72
ResNet-18	Watermark	CIFAR-10	100	100	100	100	100	100	100	100	100
		MNIST	100	100	100	100	100	100	100	100	100
		CIFAR-100	100	100	100	100	100	100	100	100	100
	Clean	CIFAR-10	0.00	0.00	0.00	0.00	0.00	0.00	0.00	0.00	0.00
		MNIST	0.00	0.00	0.00	0.00	0.00	0.00	0.00	0.00	0.00
		CIFAR-100	0.00	0.00	0.00	0.00	0.00	0.00	0.00	0.00	0.00
	Ambiguous	CIFAR-10	62.37	62.51	63.46	63.22	67.91	64.11	62.09	65.84	64.23
		MNIST	61.05	63.36	66.33	61.19	66.21	66.46	63.10	61.07	61.03
		CIFAR-100	63.29	61.93	58.17	63.22	61.49	59.51	61.35	59.89	59.06

**Table 4:** Efficiency experiment results for different watermarking methods.

Methods	Datasets	Global Iteration (s)	Watermark Embedding (s)	Watermark Verification (ms)
Clean	CIFAR-10	257.01	-	-
	MNIST	233.04	-	-
	CIFAR-100	290.09	-	-
Proposed	CIFAR-10	257.14	0.13	0.064
	MNIST	233.11	0.07	0.064
	CIFAR-100	290.17	0.08	0.062
FedIPR	CIFAR-10	361.05	104.04	0.083
	MNIST	348.84	115.80	0.074
	CIFAR-100	398.94	108.85	0.091
FedCRMW	CIFAR-10	324.75	67.74	0.073
	MNIST	293.16	60.12	0.071
	CIFAR-1006	343.61	52.70	0.077
FedTracker	CIFAR-10	314.32	57.31	0.078
	MNIST	289.75	56.71	0.072
	CIFAR-100	327.16	37.07	0.081

## Data Availability

The associated datasets “MNIST”, “CIFAR-10” and “CIFAR-100” used for demonstration are publicly accessible from “http://yann.lecun.com/exdb/mnist/, accessed on 18 March 2024 ”. The links for “CIFAR-10” and “CIFAR-100” are “https://www.cs.toronto.edu/~kriz/cifar.html, accessed on 18 March 2024”, respectively.
